# Hospitalization among vaccines for SARS-CoV-2 breakthrough infection after dose sparing strategies in Libya: A cohort study

**DOI:** 10.1371/journal.pone.0276425

**Published:** 2022-11-03

**Authors:** Mohamed Hadi Mohamed Abdelhamid, Iman Amin Almsellati, Badereddin B. Annajar, Alaa.H Abdulhamid, Hafsa Alemam, Mohammed Etikar

**Affiliations:** 1 Head of Researchs and Sciences Committees Office, National Center for Disease Control (NCDC), Tripoli, Libya; 2 Department of Cell Biology and Tissue Culture, Libyan Biotechnology Research Center (BTRC), Tripoli, Libya; 3 Primary Health Care, National Center of Disease Control (NCDC), Tripoli, Libya; 4 Department of Public Health, Faculty of Medical Technology, University of Tripoli, Tripoli, Libya; 5 Department of Cardiology, Tripoli University Hospital, Tripoli, Libya; 6 Department of Environment, Biotechnology Research Center (BTRC), Tripoli, Libya; Al-Jouf University College of Pharmacy, SAUDI ARABIA

## Abstract

SARS-CoV-2 infection is widely spread over people, from youth to the elderly. Vaccination against SARS-CoV-2 is an essential preventive measure to help end the SARS-CoV-2 pandemic. A multi-center retrospective cohort study was conducted on patients in Libya who had received single-dose licensed three different types of vaccines (Oxford/AstraZeneca, CoronaVac, or Sputnik-V) and were admitted to healthcare centers with SARS-CoV-2 infection from 30^th^ April to 15^th^ July 2021. In this study, the number of people infected with SARS-COV-2 and the mortality rate from daily reports issued by the National Centers for Disease Control of Libya (NCDC) were collected. Approximately 445000 single doses of the SARS-COV-2 vaccine were administered in Libya from April to July 2021. In corresponding, 39996 people were infected during this period. It has been found that among the people who did not receive any vaccine, the number of patients infected by SARS-COV-2 and admitted to the healthcare centers, and died was (N = 3176 patients (7.94%), and 266 (7.10%) respectively). Compared to 43 (0.10%) of those admitted to healthcare centers who had taken a single dose from one of the licensed vaccines, of which 8 patients (0.02%) died during this period. The documented 23 patients were those who admitted to healthcare centers and got vaccinated with the CoronaVac (Sinovac) vaccine. Fourteen patients received Oxford/AstraZeneca. Only 2 patients received the Sputnik V vaccine. Of the breakthrough infection cases reviewed, 8 patients died. No deaths due to breakthrough infection among Sputnik V vaccinated patients were reported. In conclusion, a single dose of the three different types of the vaccine has significantly reduced virus interpersonal transmission and also showed a decrease in the mortality rate until the tenth week in Libya. The present study demonstrates the extent of the remarkable success of the early rollout of the coronavirus national vaccination campaign.

## Introduction

SARS-CoV-2 infection is widely spread among people, from youth to elderly individuals. Mass vaccination operations to prevent coronavirus disease 2019 (COVID-19) are ensuing in 207 countries; as of May 2022, 11.8 billion vaccine doses have been administered, with 34.86 million are now administered daily. Currently, 64% of the global population had received at least one dose of a SARS-CoV-2 vaccine, and 59% are fully vaccinated [1, 2]. Interestingly, several studies have found that most hospitalizations and deaths due to SARS-CoV-2 infection were among immunocompromised individuals, persons with comorbidities, elderly individuals, and/or unvaccinated people [3–5].

Furthermore, previous studies showed that vaccines reduce the risk of SARS-CoV-2 infection, especially illness severity, among partially vaccinated people [6–9] (Table 1).

**Table 1:** The efficacy of the vaccines against symptomatic, severe, and hospitalization SARS-CoV-2 infection.

*Vaccines*	*SARS-CoV-2 infection*	*vaccines against symptoms of SARS-CoV-2 after receiving the first dose*	*Against hospitalization after receiving the first dose*
***AstraZeneca***	67%	68.7%	76%
***Sputnik-V***	80%	81%	87%
***CoronaVac***	49.6%	70%	99.2%

The NCDC of Libya reported that the first case of SARS-CoV-2 was identified in Libya on 24 March 2020 [10–12]. In addition, as reported by the World Health Organization (WHO), many countries were not very well prepared to deal with the virus; for example, Libya, Iraq, and Yemen are most vulnerable to the impact of this pandemic [13].

In Libya, the national immunization program against SARS-COV-2 started somewhat late on 10 April 2021 through 400 immunization center distributed all around the country [12]. This was mainly due to the difficulties in obtaining the vaccine by direct procurement or even through the COVAX facility, the NCDC therefore, reluctantly announced a deviation from the recommended protocol for SARS-CoV-2 vaccines by prolonging the interval between doses from 2 to 4 months, these strategies are collectively known as ‘dose-sparing’ strategies [14].

In fact, most studies suggest that a single-dose vaccine strategy might confer high efficacy against SARS-CoV-2 infection and disease severity, two studies in Scotland and England confirmed high protection rates (80%–91%) after the first dose of Pfizer or Oxford-AstraZeneca (ChAdOx1, nCoV-19/AZD1222) vaccines [3, 4]. A single dose of the Sputnik V (Gam-COVID-Vac) vaccine may be enough to elicit a strong antibody response against SARS-CoV-2 [15, 16].

Worldwide, many hospitals have recorded cases of SARS-CoV-2 infection in patients after being vaccinated; this situation is called a vaccine breakthrough infection [17, 18]. Although SARS-CoV-2 vaccines appear to be very effective against disease severity and deaths, however, in some cases may not provide full protection [19]. In the United States, the U.S. The Centers for Disease Control and Prevention (CDC) reported a total of 10,262 SARS-CoV-2 vaccine breakthrough infections from 46 U.S. states and territories as of April 30, 2021 [20]. A recent study reported that the effectiveness of the vaccines against infections decreased from 91.7% to 79.8% between 3 May and 25 July in New York [21]. Moreover, a study published by the National Healthcare Safety Network (NHSN) found that the two mRNA (nucleoside-modified) vaccines (Pfizer-BioNTech and Moderna) were effective by 74.7% in nursing home residents between March and May; however, the protection declined to 53.1% between June and July [22].

Certainly, investigating the trend of SARS-CoV-2 infection among persons who received the SARS-CoV-2 vaccine is urgently needed to support decision-making logistics such as cold chains, vaccination schedules, and follow-up. In this context, this retrospective, cohort, a multi-center study was conducted to investigate the trend of SARS-CoV-2 infection severity, hospitalization rate, and mortality for patients who received a single-dose of three different types of licensed vaccines in Libya.

## Materials and methods

### Data collection

This study was performed on patients who received single-dose a licensed three different types of vaccines and were admitted to healthcare centers with confirmed SARS-CoV-2 by repeat reverse-transcriptase polymerase chain reaction (RT–PCR) assays from 30 April to 15 July 2021. The data were collected from the patients’ medical records by intensive care physicians and healthcare center medical staff throughout 34 healthcare centers and hospitals all around the country.

### Study design

The study has been principally designed to evaluate the effect of a single-dose of the Oxford-AstraZeneca (nCoV-19/AZD1222), CoronaVac (PiCoVacc), and Sputnik V (Gam-COVID-Vac) vaccines on outcomes related to breakthrough vaccine infection. Case information was included in our study of all vaccinated and unvaccinated people admitted to the healthcare centers during the indicated period of time, no SARS-COV-2 infection was reported before the first dose of the vaccine, and the age was 16 years or older. The people who received the first dose of the SARS-CoV-2 vaccine on 10 April 2021 were at the same time tracking. Confirmed COVID-19 positive diagnosis of cases was performed according to WHO guidelines [23]. Patients who had no RT–PCR test or were their negative results were excluded. According to the severity criteria were outlined [24, 25], all included patients were admitted to healthcare centers and classified as mild, moderate, severe, or critical. All cases’ names were omitted and coded to protect their privacy. All data were reviewed by 3 physicians and then analyzed by a statistician.

Continuous variables were presented as (mean ± SD) and were compared using a two-way analysis of variance. Cases were divided into two groups (unvaccinated and vaccinated people) and used the percentage of coefficient of variation (%CV; standard deviation/mean x 100) to evaluated the relative dispersion of data. In all cases, p values <0.05 were considered statistically significant. Matched individuals were younger (median age 57 years (interquartile range [IQR], 54 to 67)) than the eligible population who were vaccinated (63 years, IQR 48 to 71). All data were processed using the Statistical Package for Social Sciences, version 25 (SPSS, Chicago, IL, USA).

### Ethics statement

The study was approved by the Ethics Committee (Libyan National committee for Biosafety and Bioethics, N: LNCBB 22–11). The protocol was previously published [23], and the study was carried out according to the Helsinki Declaration.

## Result

In this retrospective cohort study, according to the NCDC data from February 01, 2021 (W1) to July 15, 2021 (W22). Approximately 445000 single doses of the SARS-COV-2 vaccine were administered in Libya from April to July 2021. In corresponding, 39996 people were infected during this period. Among the people who did not receive any vaccine, the number of patients infected by SARS-COV-2 and admitted to the healthcare centers, and died was (N = 3176 patients (7.94%), and 266 (7.10%) respectively). Compared to 43 (0.10%) of those admitted to healthcare centers who had taken a single dose from one of the licensed vaccines (Oxford/AstraZeneca, CoronaVac, or Sputnik-V), of which 8 patients (0.02%) died during this period.

Those 23 patients who were admitted to healthcare centers and vaccinated with the CoronaVac (Sinovac) vaccine were documented. Fourteen patients received Oxford/AstraZeneca. Only two patients received the Sputnik V vaccine. Of the breakthrough infection cases reviewed, 8 patients died. No single death due to breakthrough infection among Sputnik V vaccinated patients was reported. The average age of patients admitted to healthcare centers was between 55–87 years old (Table 2).

**Table 2:** Numbers of patients who were admitted to healthcare centers or hospitals after receiving one dose of vaccine in Libya. N: Number DM: Diabetic HT: Hypertension CoronaVac: Sinovac Astra: Oxford/AstraZeneca SputV: Sputnik v (-) = Data not available.

N	Healthcare Center	N of the patient (Type of vaccines)	N of died (Type of vaccine)	Age and gender at the death of the patient (Type of diseases)
**1**	Derna	2 (CoronaVAC)	1 (Astra)	F, 70 Y (DM)
		1 (Astra)		
**2**	Marj	2 (CoronaVac)	0	-
**3**	Bayda	1 (CoronaVac)	0	-
**4**	Sabha	1 (Astra)	1 (Astra)	F, 80 Y (DM)
**5**	Tripoli 1	1 (Astra)	1 (CoronaVac)	F, 87 Y (HT)
		1 (CoronaVac)		
		1 (Sput V)		
**6**	Tripoli 2	-	-	-
**7**	Zliten	3 (Astra)	0	-
**8**	Zawiya	-	-	-
**9**	Misrata	-	-	-
**10**	Zuwara	2 (CoronaVac)	1 (CoronaVac)	M, 61 Y (HT)
		1 (Astra)		
**11**	Tagy	1 (Astra)	0	-
**12**	Tobruq	2 (CoronaVac)	1 (Corona Vac)	M, 78 Y (DM, HT)
**13**	Ghadames	2 (CoronaVac)	0	-
		1 (Astra)		
**14**	Benghazi	2 (Astra)	0	-
**15**	Shakshok	2 (CoronaVac)	1 (CoronaVac)	F, 63 Y
**16**	Msallata	3 (CoronaVac)	1 (CoronaVac)	M, 80 Y (DM)
**17**	Khoms 1	1 (CoronaVac)	0	-
		1 (Astra)		
**18**	Khoms 2	1 (Sput V)	1 (CoronaVac)	M, 64 Y (DM)
		3 (CoronaVac)		
		2 (Astra)		
**Total N of patient = 43 Total N of patient Died = 8**				

According to the results of this study, the mean ± SD patients positive testing decreased significantly (*P* ≤ 0.05) after 21 days (W13) beginning to vaccine program from (W10) 628±31.3 to 270±23 per week (*P* = 0.0015), at the same time, the mortality rate declined by the same points from 10.5±3.1 to 2.8±0.8 patients per week (*P* = 0.0012). Additionally, the Coefficient of variation (CV) between before and after vaccines are 33% and 14% respectively.

**Fig 1:**
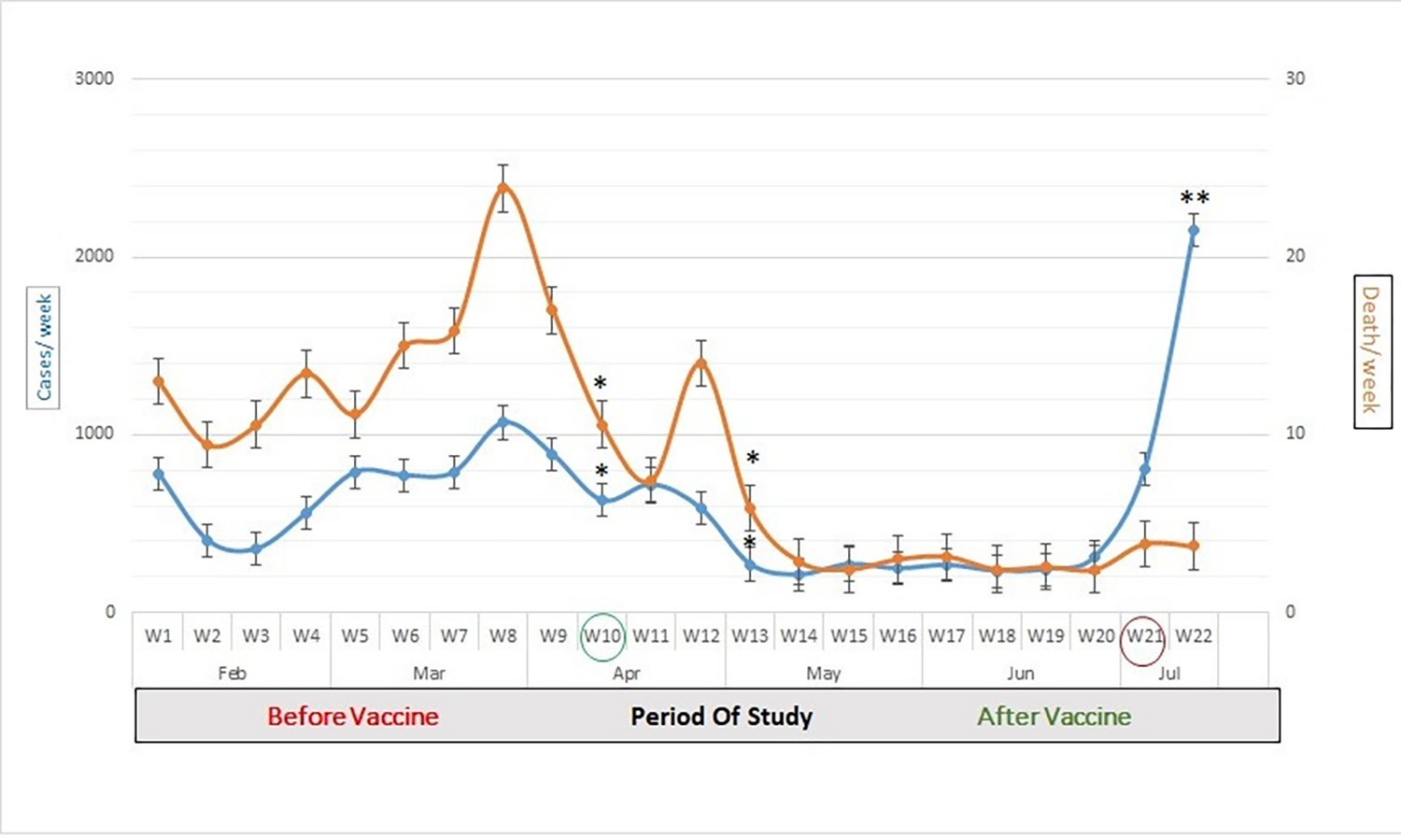
Mean of patients with confirmed SARS-COV-2 infection from February to 15 July in Libya, (*): High significant different (a p-value <0.05 was considered significant); (**): Very high significant different (a p-value <0.05 was considered significant). Green ring: start to vaccines program; Red ring: first cases with Delta variant.

**Fig 2:**
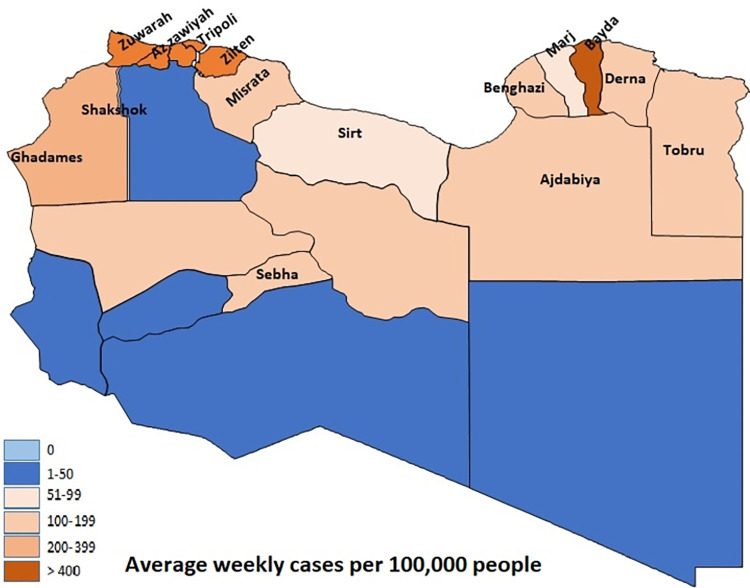
The geographic distribution of SARS-COV-2 infection in Libya before and after vaccine. Average weekly cases per 100,000 people. SARS-COV-2 infection in Libya before vaccine.

**Fig 3:**
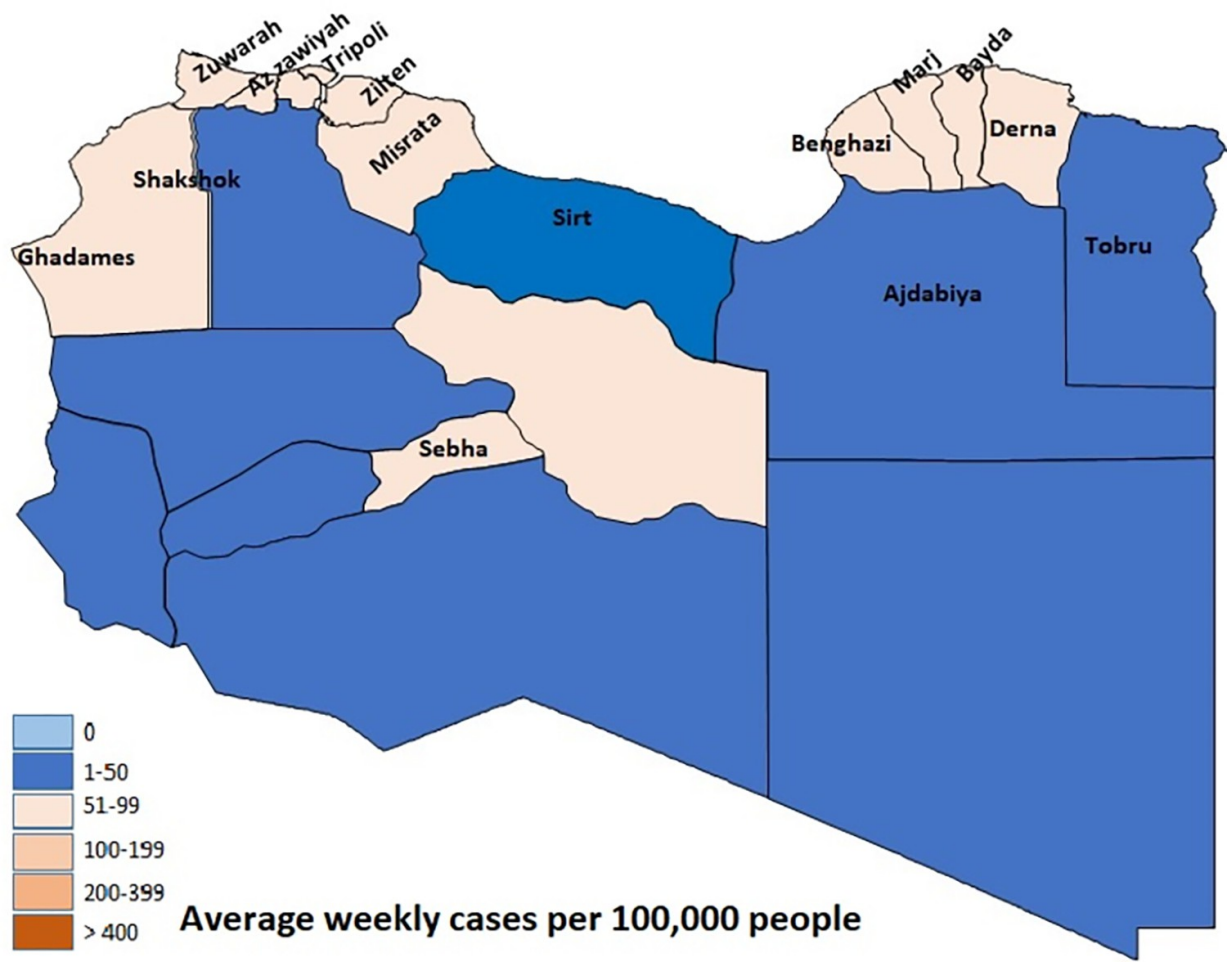
The geographic distribution of SARS-COV-2 infection in Libya before and after vaccine. Average weekly cases per 100,000 people. SARS-COV-2 infection in Libya after vaccine. Created by Microsoft, open streetMap.

Since July, the NCDC has reported initial evidence that people are being infected with the Delta variant. Whereas the mean testing positively increased nine-folds, from 228 ±0.001 the previous week to 2148 ± 0.032 cases. It recorded its highest number of cases on 12 July (W20). However, the number of patient deaths remains lower compared to W11 (5.8–3.8 patients per week) (Fig 1). In this period (W20), with delays in travel restrictions, there was a significant increase in the number of people confirmed to have SARS-CoV-2. Particularly in the western region in cities such as Zawiya, Tripoli, Shakshok, Zliten, and Misrata, due to their proximity to the Tunisian borders, the peak infection rates were recorded in Africa and the world with the Delta variant (Figs 2 and 3).

## Discussion

The SARS-COV-2 vaccination program was initiated in Libya on 10 April 2021, with free vaccinations provided to all Libyan residents in phases; priority was given to frontline healthcare and hospital workers, adults over 55, and patients with chronic underlying health conditions in all regions of the country.

In addition, A single dose strategy may be sufficient to confer immunity and protection against severe acute respiratory syndrome coronavirus 2 (SARS-CoV-2) infection at the population level, especially in low- and middle-income countries where vaccine supply remains limited. Our study period included the early part of the vaccination campaign, when the proportion of older individuals receiving the first dose of the vaccine was high. Vaccines used are Oxford/AstraZeneca, CoronaVac, and Sputnik-V. As of 15 July 2021, 445000 had been administered (around 7% of the total population) as the first dose of SARS-CoV-2 vaccines provided by the national immunization program.

United Nations International Children’s Emergency Fund (UNICEF), through COVAX, supported the Libyan government by delivering 57,600 doses on 8 April 2021(Situation et al., 2021). Additionally, as international aid, the Libyan government received approximately 200,000 doses of (Sputnik V) vaccines and 150,000 doses of Sinovac vaccine in April 2021(Mahmoud et al., 2021).

Hence, 110,000 doses of Sinovac, 175,000 doses of Oxford/AstraZeneca, and 160,000 doses of Sputnik V were administered. NCDC has recommended that the Oxford/AstraZeneca vaccine be used for people aged over 55 years old. The other vaccines (Sinovac and Sputnik V) are to be used for people aged 16 years old or older.

Moreover, due to delays in providing vaccines throughout COVAX and the countries that are producing the vaccines, the NCDC announced a deviation from the recommended protocol for SARS-CoV-2 vaccines, prolonging the interval between doses from 2 to 4 months, these strategies are collectively known as ‘dose-sparing’ strategies. This procedure has two advantages. First, a longer gap between doses may improve the long-term immune response, as demonstrated by Oxford/AstraZeneca’s vaccine (Iacobucci and Mahase, 2021). Second, a larger number of elderly individuals and people with chronic diseases will be vaccinated. In the same context, several studies Evidence tentatively that the net vaccine escape risk is lower when more hosts are vaccinated with single doses than when fewer hosts are vaccinated twice due to reduced cases [26–28]. a recent prospective cohort study from Scotland found the first dose of the Oxford-AstraZeneca vaccine was 88% (95% CI, 75 to 94) [4], Public Health England has reported that the effects of a single-dose vaccine against hospital admission for Oxford/AstraZeneca were 80%. In the second part of these studies, they found a similar vaccine effect against COVID-19 hospital admission for the Oxford/AstraZeneca vaccines after a single dose [29].

In contrast, concerns have been raised that expanding the fraction of the population that is partially immune to SARS-CoV-2 could increase of selection of vaccine-escape variants, ultimately undermining vaccine effectiveness [30]. With two-dose prime-boost mRNA vaccination, a high neutralizing antibody titer is obtained, up to 50-fold compared to a prime-only regimen. As well, the more robust the immunity in a given vaccinated, the less likely it is that the individual would experience disease or pose a transmission hazard to contacts [31, 32]. In Libya, in a preprint study, the level of IgG for SARS-COV-2 vaccines (Oxford/AstraZeneca, CoronaVac, and Sputnik-V) declined 7–15 weeks after receiving the first dose [33]. However, all these results were obtained after the delta variant entered Libya.

### Limitations of the study

The results of this study are subject to two limitations. The first limitation is that the number of reported SARS-CoV-2 vaccine breakthrough cases is substantially lower than that of all SARS-CoV-2 infections among one-dose vaccinated persons. However, many people with vaccine breakthrough infections, especially those who are asymptomatic or who experience mild illness, might not seek testing. Second, SARS-CoV-2 sequence data are available for only a small proportion of the reported cases.

## Conclusion

In the present study, one dose of the three different types of vaccines showed a decrease in infection and mortality rates. The results were very encouraging. However, all these results were obtained before the delta variant entered Libya. As SARS-CoV-2 variants continue to emerge, understanding the interplay between virus evolution and vaccine durability will be very important for revising vaccination strategies and formulations as we seek to curb the ongoing pandemic. As a recommendation, the Department of Health and NCDC must focus and make all possible efforts to get as many people vaccinated within a short period to prevent the development of new virus variants.
